# Supplementary material for: Metabolomics Profiling of Cystic Renal Disease towards Biomarker Discovery
Source: Biology (Basel). 2021 Aug 13;10(8):770. doi: 10.3390/biology10080770 (PMC8389671; doi:10.3390/biology10080770)
Supplement: Supplementary file 1 [file biology-10-00770-s001.zip › biology-1264082-supplementary.pdf]

biology MDPI

Metabolomics Profiling of Cystic Renal Disease Towards Biomarker Discovery

Dalia Sriwi[1¥], Mohamad S. Alabdaljabar[1¥], Minnie Jacob[2], Essa M. Sabi[3], Xinyun Gu[4], Ahmad Mujammami[3], Liang Li[4], Maged H Hussein, Majed Dasouki[2], Anas M. Abdel Rahman[1,2, 6*]

[1]Department of biochemistry and molecular medicine, College of Medicine, Alfaisal University, Riyadh, Saudi Arabia

[2]Metabolomics Section, Department of Clinical Genomics, Center for Genomics Medicine, King Faisal Specialist Hospital and Research Centre (KFSHRC), Riyadh, Saudi Arabia

[3]Clinical Biochemistry Unite, Department of Pathology, College of Medicine, King Saud University, Riyadh, Saudi Arabia

[4]Department of Chemistry, University of Alberta, Edmonton, AB, T6G 2R3, Canada

[5]King Faisal Specialist Hospital and Research Centre (KFSHRC), Riyadh, Saudi Arabia.

[6]Department of Chemistry, Memorial University of Newfoundland, St. John's, NL, A1B 3X7, Canada

*Correspondence to Anas Abdel Rahman,

Metabolomics Section, Department of Clinical Genomics, Center for genomics medicine, King Faisal Specialist Hospital and Research Centre (KFSHRC), MBC#03

PO Box 3354, Riyadh 11211, Kingdom of Saudi Arabia

Tel#: +966114647272 Ext. 36481

Fax#: +966114424585

Email: aabdelrahman46@kfshrc.edu.sa

¥ DS and MSA contributed equally and are first authors

Supplementary Figures:

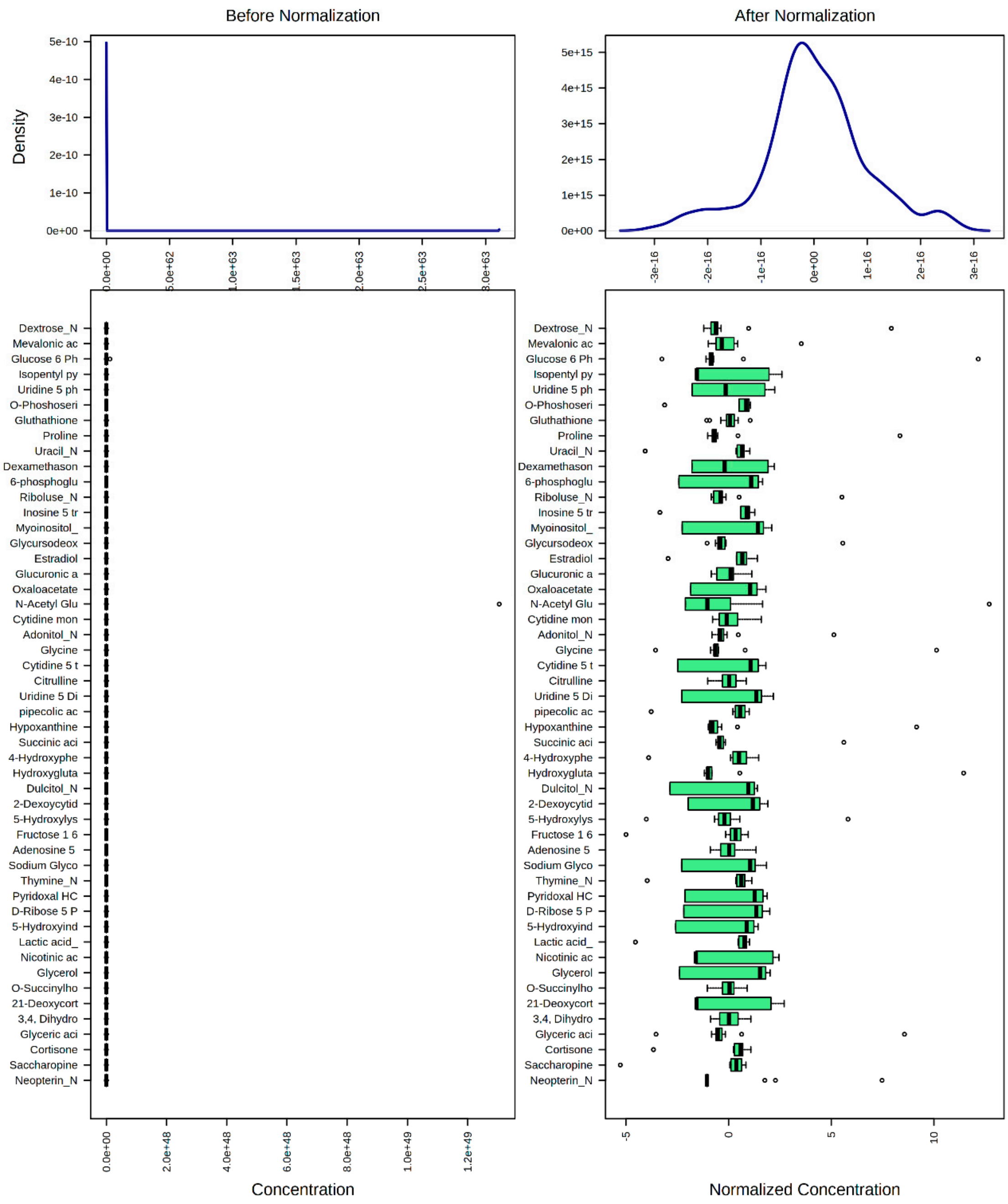

Figure S1: Normalization of data in patients with cystic renal disease (CRD) against the Ctrl groups was performed to remove systemic variations in relation to biological differences, and ensure the normal distripution.

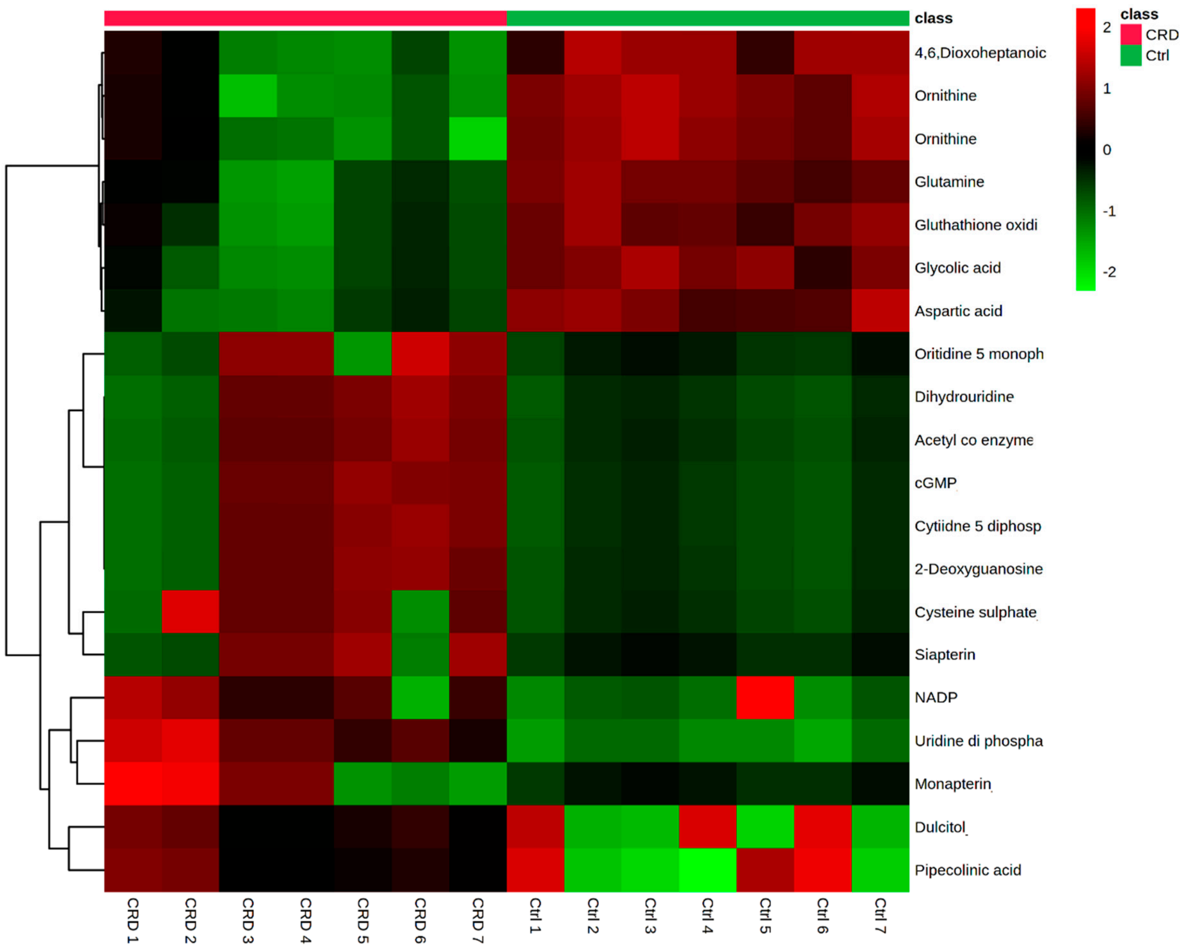

Figure S2: Heat map in DBS shows altered metabolites in patients with cystic renal disease (CRD) and Ctrl groups. The heat maps were created by entities Hierarchical clustering for the normalized data where the similarity was based on Pearson

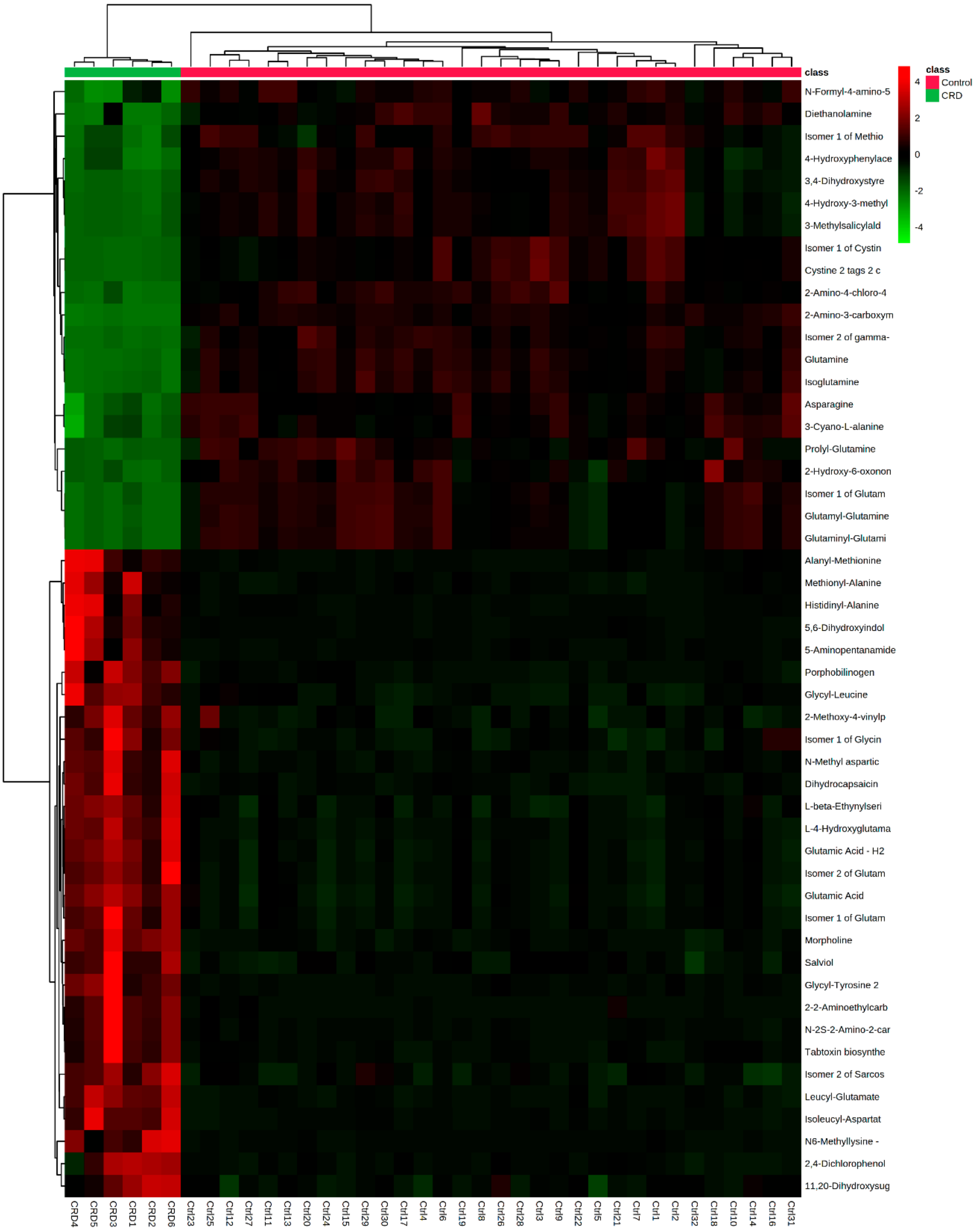

Figure S3: Heat map in serum through CIL targeted analysis shows altered metabolites in patients with cystic renal disease and controls. The heat maps were created by group Hierarchical clustering for the averaged normalized data where the similarity was based on Euclidean.

Supplementary Tables:

Table S1: Statistically significant pathways dysregulated in CRD patients compard to healthy control based on DBS metabolomics profile

Pathway Name	-log(p)	FDR	Impact
Pyrimidine metabolism	3.3018	0.041921	0.13965
Glutathione metabolism	2.8872	0.043665	0.02878
Arginine biosynthesis	2.7737	0.043665	0.06091
Nicotinate and nicotinamide metabolism	2.6821	0.043665	0.0
Alanine, aspartate and glutamate metabolism	1.8932	0.21485	0.42869
Glyoxylate and dicarboxylate metabolism	1.734	0.25829	0.03175
Purine metabolism	1.5683	0.32424	0.069
Pantothenate and CoA biosynthesis	1.3514	0.40733	0.00714
Citrate cycle (TCA cycle)	1.3104	0.40733	0.1545

Table S2: Significantly changed metabolites in CRD patients compared to healthy controls based on DBS metabolomics profile (FDRp and Fold change cut-offs are 0.05, and 2, respectively)

Name of the metabolite	HMDB	log2(FC)	-Log10(p)
Deoxyribose 5-phosphate	HMDB0001031	-2.7455	1.4857
Ornithine	HMDB0000214	-2.5939	3.3467
Succinylacetone	HMDB0000635	-1.8121	2.6264
L-Aspartic acid	HMDB0000191	-1.6616	1.5995
Taurine	HMDB0000251	-1.6095	1.3622
Taurodeoxycholic acid	HMDB0000896	-1.4678	1.4059
Glycolic acid	HMDB0000115	-1.4657	1.9758
Adenosine monophosphate	HMDB0000045	-1.2176	1.4494
D-Sedoheptulose 7-phosphate	HMDB0001068	-1.2123	1.3039
D-Mannose 1-phosphate	HMDB0006330	-1.1568	1.5243
Pantothenic acid	HMDB0000210	-1.1358	1.5353
Uridine	HMDB0000296	-1.0763	1.3512
Oxidized glutathione	HMDB0003337	-1.0392	1.6258
L-Glutamine	HMDB0000641	-1.0391	1.6987
Oxalacetic acid	HMDB0000223	1.0254	1.3932
Pipecolic acid	HMDB0000070	1.1785	1.7852
NADP	HMDB0000217	1.2378	2.2021
Galactitol	HMDB0000107	2.4893	1.9792
Sepiapterin	HMDB0000238	18.751	1.7905
L-Threoneopterin	HMDB0000727	19.025	1.7929
Acetyl-CoA	HMDB0001206	19.131	2.6147
Cysteine-S-sulfate	HMDB0000731	19.472	2.6189
Deoxyguanosine	HMDB0000085	20.25	2.6193
5,6-Dihydrouridine	HMDB0000497	20.598	2.6195
CDP	HMDB0001546	20.699	2.6206
Cyclic GMP	HMDB0001314	20.839	2.6211
NA	HMDB0000218	21.669	1.7954
Uridine 5'-diphosphate	HMDB0000295	27.773	11.405
17-Hydroxyprogesterone	HMDB0000374	23.481	1.3034
Quinolinic acid	HMDB0000232	22.37	1.3759
Fructose 6-phosphate	HMDB0000124	23.399	1.4249

Table S3: Statistically significant dysregulated pathways in CRD patients compared to healthy control based on metabolomics profiingof their serum samples.

Pathway Name	-log(p)	FDR	Impact

Aminoacyl-tRNA biosynthesis	2.64	0.18	0.00
Arginine biosynthesis	2.37	0.18	0.31
Tryptophan metabolism	1.81	0.44	0.41
Cysteine and methionine metabolism	1.63	0.47	0.31
Nitrogen metabolism	1.47	0.47	0.00
D-Glutamine and D-glutamate metabolism	1.47	0.47	0.50
Alanine, aspartate and glutamate metabolism	1.30	0.60	0.33
Tyrosine metabolism	1.23	0.62	0.22
Ubiquinone and other terpenoid-quinone biosynthesis	1.14	0.68	0.00

Table S4: Significantly changed metabolites in CRD patients compared to healthy control based on their serum metabolic profile. (FDRp and Fold change cut-offs are 0.05, and 2, respectively)

Name of the metabolite	FC	log2(FC)	p-value	-Log10(p)
2-Amino-3-carboxymuconate semialdehyde	0.07	-3.93	3.3E-30	29.48
Glutamine	0.14	-2.80	2.63E-23	22.58
Gamma-Amino-gamma-cyanobutanoic acid	0.19	-2.38	3.33E-22	21.48
Cystine	0.13	-2.98	9.95E-21	20.00
Isoglutamine	0.23	-2.13	5.35E-20	19.27
Morpholine	3.28	1.71	2.46E-18	17.61
Leucyl-Glutamate	4.64	2.21	4.55E-18	17.34
Glutamyl-Glutamine	0.09	-3.46	7.31E-18	17.14
2-Amino-4-chloro-4-pentenoic acid	0.12	-3.09	4.68E-17	16.33
Glutaminyl-Glutamic acid	0.10	-3.29	7.8E-17	16.11
Isoleucyl-Aspartate	5.90	2.56	1.16E-16	15.93
3,4-Dihydroxystyrene	0.19	-2.38	1.35E-16	15.87
Glutamyl-Glutamine	0.11	-3.13	2.28E-16	15.64
Prolyl-Glutamine	0.17	-2.56	3.37E-16	15.47
3-Methylsalicylaldehyde	0.22	-2.17	6.54E-16	15.18
4-Hydroxy-3-methylbenzaldehyde	0.21	-2.27	7.07E-16	15.15
Glycyl-Tyrosine	9.39	3.23	1.17E-15	14.93
5,6-Dihydroxyindole -	7.69	2.94	1.45E-15	14.84
Glycyl-Phenylalanine	0.10	-3.36	1.66E-15	14.78
Serotonin	0.04	-4.61	2.41E-15	14.62

L-4-Hydroxyglutamate semialdehyde	4.08	2.03	8.75E-15	14.06
5-Hydroxy-L-tryptophan	0.12	-3.03	1.42E-14	13.85
N6-Methyllysine	9.46	3.24	6.04E-14	13.22
N-Methyl aspartic acid	2.50	1.32	7.48E-14	13.13
4-Hydroxy-2,6-dimethylaniline	0.14	-2.83	9.02E-14	13.05
2-Hydroxy-6-oxonona-2,4-diene-1,9-dioic acid	0.31	-1.71	1.75E-13	12.76
Porphobilinogen	6.06	2.60	1.81E-13	12.74
5-Aminopentanamide	5.91	2.56	3.01E-13	12.52
S-Glutathionyl-L-cysteine	0.11	-3.15	3.78E-13	12.42
Dihydrocapsaicin	4.37	2.13	7.66E-13	12.12
4-Chloro-L-lysine	0.18	-2.48	1.24E-12	11.91
Histidinyl-Alanine	11.84	3.57	4.33E-12	11.36
N-2S-2-Amino-2-carboxyethyl-L-glutamic acid	10.03	3.33	9.44E-12	11.03
4-Hydroxyphenylacetaldehyde	0.35	-1.52	9.44E-12	11.03
Tabtoxin biosynthesis intermediate	5.81	2.54	1.03E-11	10.99
Methylguanidine	5.30	2.41	1.05E-11	10.98
Lysyl-Threonine	11.76	3.56	1.67E-11	10.78
Glutamic Acid	4.13	2.04	1.79E-11	10.75
Alanyl-Methionine	18.69	4.22	2.17E-11	10.66
Diethanolamine	0.32	-1.63	2.41E-11	10.62
Methionyl-Alanine sulfoxide	5.39	2.43	4.72E-11	10.33

Glutamic Acid	3.50	1.81	5.59E-11	10.25
Methionine	0.32	-1.63	6.54E-11	10.19
L-beta-Ethynylserine	2.70	1.43	7.06E-11	10.15
Glycyl-Leucine	3.67	1.87	8.81E-11	10.06
2-2-Aminoethylcarbamoylmethyl-2-hydroxybutanedioic acid	27.63	4.79	1.6E-10	9.80
7-Carboxy-7-carbaguanine	0.34	-1.55	2.1E-10	9.68
Phenylalanyl-Glutamate	0.16	-2.64	3.44E-10	9.46
Kanosamine 6-phosphate	0.35	-1.50	4.15E-10	9.38
Tyrosine 2 tags 2 charges	0.38	-1.41	4.93E-10	9.31
2,6-Diamino-4-hydroxy-5-N-methyl formamidopyrimidine	0.37	-1.42	6.49E-10	9.19
Asparagine	0.36	-1.49	9.23E-10	9.03
N-Formyl-4-amino-5-aminomethyl-2-methyl pyrimidine	0.42	-1.24	1.53E-09	8.81
Seryl-Threonine	0.29	-1.78	1.67E-09	8.78
Methionine	0.33	-1.61	1.82E-09	8.74
Xanthine	0.34	-1.56	1.94E-09	8.71
Cysteinyl-Glycine dimer	0.34	-1.56	2.65E-09	8.58
2-Methoxy-4-vinylphenol	2.49	1.31	3.45E-09	8.46
Aspartyl-Phenylalanine	0.16	-2.66	4.02E-09	8.40
Phenylalanyl-Serine	0.20	-2.31	4.45E-09	8.35
Phenylalanyl-Tryptophan	0.30	-1.72	5.87E-09	8.23
Tryptophan	0.45	-1.14	9.42E-09	8.03
Guanidoacetic acid	0.49	-1.02	9.48E-09	8.02

L-2-Amino-6-oxoheptanedioic acid	0.27	-1.90	9.95E-09	8.00
Isomer 2 of Sarcosine	2.54	1.35	1.64E-08	7.79
4-Hydroxybenzoic acid	0.44	-1.17	4E-08	7.40
3-Cyano-L-alanine	0.43	-1.22	4.65E-08	7.33
Alanyl-Proline	4.22	2.08	5.3E-08	7.28
p-Coumaroylagmatine	3.72	1.90	5.97E-08	7.22
Isoleucyl-Threonine	26.68	4.74	6.21E-08	7.21
Phenylalanyl-Leucine	0.26	-1.92	7.79E-08	7.11
Phenylalanyl-Phenylalanine	0.33	-1.59	1.24E-07	6.91
Guaiacol	0.33	-1.58	1.7E-07	6.77
Glycyl-Valine	0.04	-4.61	1.84E-07	6.74
Alanyl-Glutamic acid	0.42	-1.25	2.1E-07	6.68
Alanyl-Serine	0.18	-2.51	2.54E-07	6.60
Maltol	0.31	-1.71	3.03E-07	6.52
Isomer 1 of 7,8-Dihydroxanthopterin	0.42	-1.27	3.18E-07	6.50
Threoninyl-Lysine	10.41	3.38	3.87E-07	6.41
Homoarginine	0.35	-1.51	3.96E-07	6.40
Glutamyl-Methionine	0.40	-1.34	3.96E-07	6.40
Uridine	0.43	-1.22	4.14E-07	6.38
Alanyl-Alanine	3.28	1.71	4.26E-07	6.37
7,8-Diaminononanoic acid	2.62	1.39	4.41E-07	6.36
Uridine	0.43	-1.21	6.39E-07	6.19
Glycyl-Histidine	6.18	2.63	6.46E-07	6.19
Valyl-Glycine	2.39	1.26	7.34E-07	6.13
11,20-Dihydroxysugiol	2.32	1.21	1.02E-06	5.99

Valyl-Valine	2.47	1.30	1.51E-06	5.82
4-Formylsalicylic acid	0.45	-1.15	1.74E-06	5.76
Prolyl-Glutamate	3.16	1.66	1.99E-06	5.70
4-Methylene-L-glutamic acid	31.89	5.00	2.44E-06	5.61
Histidinyl-Lysine	6.87	2.78	2.99E-06	5.52
Glutamyl-Tyrosine	0.18	-2.50	3.16E-06	5.50
Iminodiacetic acid	2.73	1.45	3.95E-06	5.40
2,4-Dichlorophenol	4.44	2.15	7.89E-06	5.10
N2-Succinyl-L-ornithine	2.44	1.29	7.97E-06	5.10
Leucyl-Valine	2.02	1.01	8.64E-06	5.06
5-Aminopentanoic acid	2.07	1.05	9.27E-06	5.03
Arginine	0.41	-1.30	0.000014	4.94
Homomethionine	2.14	1.10	0.0000114	4.94
Asparaginyl-Lysine 2	2.04	1.03	0.000018	4.74
5-Aminopentanoic acid	2.01	1.01	0.0000199	4.70
5-L-Glutamyl-taurine	3.54	1.82	0.0000238	4.62
Lysyl-Isoleucine	3.20	1.68	0.0000262	4.58
Isoleucyl-Glycine	2.05	1.04	0.0000305	4.52
Gamma-Glutamylglutamic acid	3.23	1.69	0.000033	4.48
Seryl-Asparagine	3.35	1.75	0.0000364	4.44
Isoleucyl-Alanine	2.17	1.12	0.0000381	4.42
Salicylic acid	4.91	2.30	0.0000513	4.29
Valyl-Alanine	2.29	1.20	0.0000617	4.21
Isomer 1 of Arginine	0.45	-1.14	0.0000633	4.20
Isomer 2 of 5-Aminopentanoic acid	2.01	1.00	0.0000729	4.14

N6-Acetyl-LL-2,6-diaminoheptanedioic acid	2.37	1.25	0.0000777	4.11
Aspartyl-Alanine	2.73	1.45	0.0000791	4.10
3-3,4-Dihydroxyphenyllactic acid -	6.63	2.73	0.0000804	4.09
Tyrosyl-Asparagine	0.47	-1.08	0.0000908	4.04
Glutamyl-Glycine	0.39	-1.37	0.0001175	3.93
Asparaginyl-Proline	2.75	1.46	0.0001469	3.83
Glycyl-Arginine	3.00	1.58	0.0001476	3.83
N-L-Argininosuccinic acid	2.12	1.08	0.0001543	3.81
Calystegin A3	2.78	1.48	0.0001554	3.81
gamma-Glutamyl-beta-cyanoalanine	0.09	-3.48	0.0001984	3.70
Asparaginyl-Phenylalanine	4.15	2.05	0.0002966	3.53
4-Hydroxyphenylacetylglutamic acid	2.84	1.51	0.0003651	3.44
Asparaginyl-Isoleucine	2.05	1.03	0.0003828	3.42
Homogentisic acid	0.47	-1.08	0.0003844	3.42
Cystathionine	2.46	1.30	0.0004164	3.38
S-Adenosylhomocysteine	2.14	1.10	0.0004662	3.33
Isomer 1 of Isoleucine	2.48	1.31	0.0004985	3.30
Seryl-Serine	2.74	1.45	0.0004994	3.30
1-Methylhistidine	2.10	1.07	0.0004994	3.30
3-Aminoisobutanoic acid	2.46	1.30	0.000512	3.29
Neuraminic acid	2.17	1.12	0.0005696	3.24
2,4-Diaminotoluene	0.42	-1.26	0.0006235	3.21

4-Hydroxy-3-methylbenzoic acid	2.45	1.29	0.0006811	3.17
Isomer 1 of Leucyl-Aspartate	3.40	1.77	0.0007171	3.14
Phenylalanyl-Arginine	3.29	1.72	0.0007541	3.12
N-Hydroxy-L-phenylalanine	0.43	-1.20	0.0007628	3.12
L-gamma-Glutamyl-3R-L-beta-ethynylserine	4.10	2.03	0.0010939	2.96
Uracil	3.50	1.81	0.0011447	2.94
Lysyl-Methionine	2.60	1.38	0.0011569	2.94
Homocitrulline	2.68	1.42	0.0012446	2.91
3-Aminoisobutanoic acid	2.34	1.23	0.0012446	2.91
L-Pipecolic acid	0.45	-1.15	0.0012929	2.89
Seryl-Aspartic acid	2.70	1.43	0.0013021	2.89
Leucyl-Arginine	2.83	1.50	0.0014207	2.85
Histidinyl-Tryptophan	2.59	1.37	0.0015907	2.80
Tyrosyl-Aspartate	2.59	1.38	0.0016086	2.79
Guanosine	0.12	-3.00	0.0019267	2.72
Ophthalmic acid	3.76	1.91	0.0020433	2.69
Isoleucyl-Valine	3.16	1.66	0.0022256	2.65
Theophylline	0.34	-1.56	0.0023515	2.63
Homovanillic acid	2.98	1.58	0.0024418	2.61
D-Lysopine	2.30	1.20	0.0024418	2.61
Valyl-Phenylalanine	0.43	-1.21	0.0025074	2.60
gamma-Glutamylalanine	2.30	1.20	0.0025099	2.60
Isoleucyl-Phenylalanine	7.91	2.98	0.0032487	2.49
Phenylalanyl-Aspartate	2.41	1.27	0.0036135	2.44
Threoninyl-Glutamine	0.48	-1.06	0.0036149	2.44

Glycyl-Proline	4.88	2.29	0.0061695	2.21
Leucyl-Threonine	2.01	1.01	0.013516	1.87
Ascorbic acid	2.70	1.43	0.013557	1.87
Glutamyl-Leucine	2.85	1.51	0.014624	1.83
1-Phenylethylamine	4.17	2.06	0.015548	1.81
Isomer 1 of 4-Hydroxy-L-tryptophan	2.08	1.06	0.017426	1.76
Tryptophyl-Asparagine	3.92	1.97	0.017747	1.75
Lochnericine	0.29	-1.80	0.0273	1.56
Salicyluric acid	3.15	1.65	0.027782	1.56
Leucyl-Isoleucine	2.01	1.01	0.03122	1.51
Isomer 2 of 6-Amino-2-oxohexanoic acid	2.24	1.17	0.035817	1.45
4-Methylene-L-glutamine	2.43	1.28	0.041034	1.39
Histidinyl-Aspartate	2.49	1.32	0.043132	1.37
2-Descarboxy-cyclo-dopa	3.16	1.66	0.045603	1.34
2-Aminomuconic acid	2.40	1.26	0.048602	1.31
2-Aminobenzoic acid	3.06	1.61	0.048699	1.31

Table S5: ROCs in DBS of CRD patients

Name	AUC	T-tests	Log2 FC

Isoleucine	0.960	1.07E-06	-3.774
Asparagine	0.952	1.85E-04	-9.592
Gama Amino Butyric	0.944	5.71E-05	-1.244
Leucine	0.944	1.82E-06	-3.722
Uridine di phosphate	0.944	1.66E-08	5.775
Threonine	0.937	1.96E-05	-3.241
2,3,Pyridinedicarboxylic acid	0.937	2.12E-04	6.026
Tyrosine	0.929	2.47E-06	-5.832
Ornithine	0.929	5.52E-05	-2.827
Histidine	0.921	9.70E-06	-4.441
N-Acetyl Glucosamine 1 phosphate	0.897	5.67E-05	5.480
Ornithine	0.889	0.0039036	-3.137
Niacinamide	0.881	0.0011873	-4.096
Progesterone	0.873	0.0010556	-1.478
Mannitol	0.873	0.069393	3.167
Dulcitol	0.869	3.94E-04	3.435
Saccharopine	0.861	0.036475	-3.089
Homovanillic acid	0.857	0.0017076	-0.541
Malonic acid	0.857	0.027611	-1.860
Cytidine	0.857	5.95E-04	-2.317
Orotic acid	0.857	0.0094314	-0.406
2-Deoxyribose 5 phosphate	0.853	1.13E-04	-4.610
2-ketobutyric acid	0.849	0.0017183	-3.537
Isoxanthopterin	0.841	8.33E-04	-1.703
Nicotinic acid mononucleotide	0.837	0.0078698	4.751
cGMP	0.833	5.94E-05	2.493
Cytiidne 5 diphosphate	0.833	5.58E-05	2.563

Pipecolinic acid	0.825	0.0033065	5.262
Diydhyrouracil	0.825	0.015533	-0.620
Alpha keto glutaric acid	0.817	0.11345	-1.055
Mevalonic acid 5 phosphate	0.810	0.029023	-2.455
4,6,Dioxoheptanoic acid	0.802	0.021109	-2.011
4-Hydroxyphenylpyruvic acid	0.802	0.023305	-2.030
21-Deoxycortisol	0.802	0.0043796	0.924
Nicotinamide ribotide	0.794	0.010658	3.364
Aminoadipic	0.790	0.0027914	2.322
5-Hydroxylysine HCL	0.786	0.013782	2.848
Thymidine	0.786	0.0068864	2.690
Kynurenine	0.786	0.004125	-1.171
17 Alpha hydroxyprogesterone	0.782	0.03639	2.881
Thymine	0.782	0.0080165	-2.058
Oxaloacetate	0.778	0.0059765	0.093
N-Acetylspermine	0.778	0.15599	-0.707
2-Deoxyguanosine	0.778	6.76E-04	1.949

Table S6: ROCs in serum of CRD patients

Name	AUC	T-tests	Log2 FC
2,6-Diamino-4-hydroxy-5-N-methylformamidopyrimidine	1	1.3242E-10	1.303
2-2-Aminoethylcarbamoylmethyl-2-hydroxybutanedioic acid	1	9.8386E-12	-4.847
2-Amino-3-carboxymuconate semialdehyde	1	5.412E-33	3.814
2-Amino-4-chloro-4-pentenoic acid	1	1.1727E-18	3.285
2-Hydroxy-6-oxonona-2,4-diene-1,9-dioic acid	1	3.9638E-14	1.550
3,4-Dihydroxystyrene	1	3.7147E-18	2.234
3-Cyano-L-alanine	1	3.3714E-08	1.264
3-Methylsalicylaldehyde	1	3.1729E-17	1.993
4-Chloro-L-lysine	1	2.4036E-13	2.307
4-Hydroxy-2,6-dimethylaniline	1	1.2843E-14	2.397
4-Hydroxy-3-methylbenzaldehyde	1	3.3595E-17	2.113
4-Hydroxyphenylacetaldehyde	1	1.6169E-12	1.433
5,6-Dihydroxyindole	1	4.1314E-17	-2.726
5-Aminopentanamide	1	1.7045E-14	-2.429
5-Hydroxy-L-tryptophan	1	1.8997E-15	2.927
7,8-Diaminononanoic acid	1	1.5393E-08	-1.313
Alanyl-Alanine	1	3.3577E-08	-1.770
Alanyl-Methionine	1	1.7603E-12	-3.930
Alanyl-Proline	1	2.2861E-09	-1.612
Asparagine	1	4.1142E-10	1.536
Cystine	1	1.3943E-21	2.831
Deethylatrazine	1	8.6555E-12	0.714
Dihydrocapsaicin	1	9.2822E-15	-2.213
Glutamic Acid	1	2.5446E-13	-2.192
Glutamine	1	7.6693E-25	2.647

Glutamyl-Glutamine	1	4.06E-19	3.386
Glycyl-Leucine	1	2.6662E-12	-1.972
Glycyl-Phenylalanine	1	1.5604E-16	3.132
Glycyl-Tyrosine	1	1.9258E-17	-3.213
Histidinyl-Alanine	1	1.6416E-13	-2.723
Isoglutamine	1	5.8087E-21	1.976
Isoleucyl-Aspartate	1	2.9558E-19	-2.570
Isomer 1 of Cystine	1	1.0797E-21	2.814
Isomer 1 of Glutamic Acid	1	4.9158E-13	-2.149
Isomer 1 of Glutamyl-Glutamine	1	2.0405E-17	3.123
Isomer 1 of Methionine	1	1.5993E-11	1.566
Isomer 1 of Phenylalanine	1	1.157E-11	0.720
Isomer 2 of gamma-Amino-gamma-cyanobutanoic acid	1	9.7851E-24	2.207
Isomer 2 of Glutamic Acid	1	7.5531E-13	-2.102
Isomer 2 of Sarcosine	1	2.5355E-10	-1.506
Kanosamine 6-phosphate	1	1.3475E-10	1.359
L-4-Hydroxyglutamate semialdehyde	1	2.8515E-17	-2.152
L-beta-Ethynylserine	1	8.4373E-13	-1.584
Leucyl-Glutamate	1	2.7977E-21	-2.310
Lysyl-Threonine	1	1.0627E-12	-2.901
Methionyl-Alanine sulfoxide	1	3.1491E-12	-2.302
Methylguanidine	1	2.6725E-13	-2.234
Morpholine	1	2.6124E-22	-1.467
N6-Methyllysine	1	1.0228E-15	-3.094
N-2S-2-Amino-2-carboxyethyl-L-glutamic acid	1	1.6339E-13	-3.347
N-Methyl aspartic acid	1	9.4909E-17	-1.421
Phenylalanine	1	7.8451E-12	0.751

Phenylalanyl-Phenylalanine	1	5.6324E-08	1.378
Porphobilinogen	1	1.5372E-15	-2.647
Prolyl-Glutamine	1	3.7101E-17	2.367
Salviol	1	3.0775E-12	-0.992
Sarcosine	1	1.5837E-10	-0.873
Serotonin	1	3.6956E-16	4.172
eryl-Threonine	1	1.0702E-09	1.696
Tabtoxin biosynthesis	1	1.6566E-13	-2.571
Tyrosine	1	9.3861E-11	1.306
7-Carboxy-7-carbaguanine	0.995	8.2794E-11	1.408
Glycyl-Valine	0.995	5.2643E-08	3.714
Guaiacol	0.995	5.7539E-08	0.731
Homoarginine	0.995	2.0214E-07	1.400
Isomer 1 of Glycine	0.995	2.5239E-11	-1.011
Isomer 1 of Iminodiacetic acid	0.995	2.2635E-07	-1.661
Maltol	0.995	2.0002E-07	1.819
Phenylalanyl-Glutamate	0.995	7.0729E-11	2.432
Prolyl-Glutamate	0.995	1.0531E-07	-1.741
S-Glutathionyl-L-cysteine	0.995	4.8284E-14	3.698
11,20-Dihydroxysugiol	0.99	5.4721E-08	-1.314
Guanidoacetic acid	0.99	7.2084E-09	0.896
Isomer 1 of 3-Hydroxymandelic acid	0.99	3.483E-08	-0.706
L-Norleucine	0.99	7.0961E-08	0.648
Methionine	0.99	5.6423E-10	1.582
Phenylalanyl-Serine	0.99	2.4646E-09	2.189
Serine	0.99	8.4276E-08	0.897
Uridine	0.99	8.2036E-07	1.026

2-Methoxy-4-vinylphenol	0.984	4.2727E-11	-1.471
4-Hydroxybenzoic acid	0.984	1.3871E-08	1.002
Alanyl-Serine	0.984	1.3652E-07	2.395
Phenylalanyl-Tryptophan	0.984	2.2433E-09	1.823
Thyroxine	0.984	3.2451E-09	0.850
Tryptophan	0.984	4.5283E-09	1.021
2R,4S-2,4-Diaminopentanoic acid	0.979	9.1234E-10	-0.881
Aminoacrylic acid	0.979	1.234E-07	0.896
